# Prevalence and antibiogram of bacteria causing urinary tract infection among patients with chronic kidney disease

**DOI:** 10.1515/med-2023-0824

**Published:** 2023-10-19

**Authors:** Tika Bahadur Thapa, Sushant Pokhrel, Anit Lamichhane, Vinay Kumar Singh, Ojaswee Shrestha, Manisha Sapkota, Puspa Raj Khanal

**Affiliations:** Department of Laboratory Medicine, Manmohan Memorial Institute of Health Sciences, Soalteemode, Kathmandu, Nepal; Department of Pathology, Sumeru Hospital Pvt Ltd, Dhapakhel, Lalitpur, Nepal

**Keywords:** urinary tract infection, chronic kidney disease, antimicrobial resistance, Nepal

## Abstract

Identifying and appropriately managing urinary tract infections (UTIs) among chronic kidney disease (CKD) patients are essential to reduce further disease complications and economic burden. Hence, this study aims to determine the prevalence of UTIs among CKD patients and study the antibiogram of the bacterial isolates. Four hundred eighty-two clean catch midstream urine samples were collected from CKD patients during the study period. The samples were cultured, and bacteria were isolated using standard microbiological techniques. Antibiotic susceptibility testing was performed by the Kirby–Bauer disc diffusion method following the Clinical and Laboratory Standards Institute guidelines. Of the 482 CKD patients, 15.8% were culture positive, and the majority was elderly aged group population. Most bacterial isolates were *Escherichia coli* 50%, followed by *Pseudomonas aeruginosa* 15.80%, *Enterococcus* species 15.80%, and *Klebsiella pneumoniae* 11.84%. The majority of bacteria were found to be resistant to beta-lactam antibiotics, ampicillin (94.67%), ceftriaxone (89.04%), cefotaxime (87.5%), and ceftazidime (84.0%), while polymyxin, colistin, vancomycin, meropenem, and imipenem were the most sensitive antibiotics. In our study, higher levels of antibiotic resistance were observed among urinary isolates. Therefore, our findings suggest clinicians to choose better antibiotic options to treat UTIs among CKD patients.

## 1 Introduction

Urinary tract infection (UTI) is the most frequent bacterial infection in clinical practice worldwide [1]. In addition, UTI is common in females, immunocompromised patients, diabetic population, incontinence with an indwelling catheter, and advanced age [2]. UTI is considered the most common and costly health-related problem worldwide. Gram-negative bacteria (GNB), Gram-positive bacteria (GPB), and some fungi are involved in the development of UTIs [3]. The organisms responsible for UTI are Enterobacteriaceae – *Escherichia coli, Klebsiella*, *Proteus*, *Pseudomonas*, and *Enterobacter* spp. and gram-positive cocci such as *Staphylococcus saprophyticus* and *Enterococcus* spp. [4].

The main problem with current antibiotic therapies is the rapid emergence of resistance in hospitals and the community [5]. As a result, UTI patients may suffer from poor outcomes of their treatments due to the emergence of antimicrobial resistance (AMR). Resistance toward antibiotics could be natural (intrinsic) or due to genetic alterations [6]. AMR takes place when microorganisms show resistance to antimicrobial drugs that were previously effective. In the long term, AMR may cause an economic burden for patients, as it will raise healthcare costs through more extended hospital stays, leading to morbidity and mortality [7]. International guidelines for treating uncomplicated UTIs and pyelonephritis recommend various agents, such as nitrofurantoin, trimethoprim-sulfamethoxazole, fosfomycin, fluoroquinolones, and beta-lactams. However, an alarming level of AMR develops in UTI pathogens due to the indiscriminate and widespread use of antibiotics resulting in multi- and often pan-resistance. Therefore, it is essential to precisely monitor and manage the spread of resistance to lower the AMR’s rising rate [8].

Chronic kidney disease (CKD) is increasingly recognized as a global public health problem. Patients with CKD and kidney failure are at high-risk for infectious complications, similar to patients with other types of acquired immune deficiencies or those treated with immunosuppressive drugs [9]. CKD patients are three times more at risk of infections than the general population [5]. Also, prolonged hemodialysis may compromise their immune system, making them vulnerable to disease, including UTIs. As a result, CKD may lead to recurrent UTIs and retrospective infection of the kidneys. In addition, chronic renal failure is a risk factor for developing UTIs due to metabolic disorders resulting in secondary immune alterations affecting many resistant components [10,11].

Moreover, in patients with chronic renal failure, UTIs occur frequently after kidney transplantation [2,9]. However, there are limited studies on the clinical characteristics of UTI in CKD patients from Nepal. When administering empirical antibiotic therapy, knowledge of the common organisms and their antibiotic profiles is essential. Therefore, this study aimed to determine the prevalence of bacteria causing UTIs and study their AMR pattern toward commonly used antibiotics among patients with different stages of CKD.

## 2 Materials and methods

### 2.1 Study design and population

We conducted a cross-sectional study over 1 year, from July 2019 to July 2020, among CKD patients who visited the Sumeru Hospital Lalitpur, Nepal. Estimated glomerular filtrate rate (GFR) was calculated according to the chronic kidney disease epidemiology collaboration (CKD-EPI) calculation formula [12], and different stages were categorized according to American Kidney Association [13]. Informed consent was given by all patients.

#### 2.1.1 Inclusion criteria

Patients having symptoms of UTI (painful urination, frequency or urgency of urination, and fever).

#### 2.1.2 Exclusion criteria

Patients who have undergone renal transplantation, those on immunosuppressive therapy for other medical illnesses, and those who have taken antibiotics within 48 h before the urine sample collection.

### 2.2 Bacteriological identification

Clean-catch midstream urine specimens were collected using leak-proof sterile plastic containers. Samples were inoculated onto a cysteine-lactose-electrolyte deficient medium using calibrated wire loop (0.001 mL) (CLED, HiMedia, India). Colonies were counted after incubation at 37°C for 18–24 h to identify significant growth. Colony counts yielding bacterial growth of greater or equal to 10^5^ colony-forming units (CFU)/mL were essential for bacteriuria. Bacteria were identified using colony characteristics, gram staining, and biochemical tests following standard procedure. Standard strains of *E. coli* (ATCC 25922), *Staphylococcus aureus* (ATCC 25923), and *Pseudomonas aeruginosa* (ATCC 27853) were used as reference strains for culture and sensitivity testing.

### 2.3 Purity plate

The purity plate was employed to ensure that the inoculation used for the biochemical test was pure culture and to see if the biochemical test was processed under septic conditions. The 4 h inoculated inoculums prepared for the biochemical test were inoculated on one-half of the nutrient agar just before proceeding for the biochemical test. The other half of the same nutrient agar will be inoculated soon after completing the biochemical test. The plate was then incubated at 37°C for 18–24 h. The growth of the same organism in both the pre- and post-inoculated portion of the plate was an indication of the maintenance of aseptic condition throughout the experiment.

### 2.4 Antibiotic susceptibility testing

Antibiotic susceptibility testing was performed by the Kirby–Bauer disc diffusion method on all significant isolates according to the Clinical and Laboratory Standards Institute guidelines [14]. Individual colonies were suspended in 5 mL of normal saline to make 0.5 McFarland standards to standardize the inoculum size. The suspensions were distributed over the entire surface of Muller Hinton agar (HiMedia, India) using sterile swabs. The antibiotic discs were placed by sterile forceps and incubated at 37°C for 18–24 h aerobically.

### 2.5 Statistical analysis

The data were collected and analyzed for results using Statistical Package for the Social Sciences (SPSS) version 23 (IBM Corp., USA). Descriptive statistics were carried out, such as frequency, percentage, and mean, in the SPSS. The Pearson Chi-square test assessed the relationship between the patient’s demographic variables and the growth of pathogens. *p*-value <0.05 was considered statistically significant.

## 3 Results

Of the 2,595 urine samples requested for culture in the microbiology department, 18.57% (482/2,595) were from CKD patients. Among the 482 CKDs, 15.8% (76/482) showed bacterial culture positivity. Among the total participants, 51.7% (249/482) were female and 48.3% (233/482) were males. The mean patients’ age (mean ± SD) was 52.73 ± 17.82 years (ranging from 9 to 96 years).

### 3.1 Demographic characteristics of CKD patients

The majority of CKD patients were from the age group 46–60 years (26.3%), followed by 61–75 years (25.9%) and 31–45 years (24.5%) (Table 1). Similarly, most patients were from CKD stage 5 (35.7%), which is followed by stage 2 (18.7%) and stage 3 (16.4%) (Table 1).

**Table 1:** Demographic characteristics of CKD patients (*N* = 482)

Characteristics	CKD stages, *n* (%)						*p*-Value
	Total	Stage 1	Stage 2	Stage 3	Stage 4	Stage 5	
**Age group**							
<15 years	8 (1.7)	0	0	1 (1.2)	1 (1.2)	6 (3.5)	0.000*
15–30 years	49 (10.2)	5 (7.8)	13 (14.5)	9 (11.4)	3 (3.8)	19 (11)	
31–45 years	118 (24.5)	37 (57.8)	28 (31.1)	12 (15.2)	10 (12.9)	31 (18)	
46–60 years	127 (26.3)	16 (25)	21 (23.3)	16 (20.3)	23 (29.8)	51 (29.6)	
61–75 years	125 (25.9)	6 (9.4)	21 (23.3)	32 (40.5)	21 (27.3)	45 (26.2)	
>75 years	55 (11.4)	0	7 (7.8)	9 (11.4)	19 (24.7)	20 (11.6)	
**Gender**							
Male	233 (48.3)	38 (59.4)	41 (45.6)	32 (40.5)	48 (62.3)	74 (43)	0.010*
Female	249 (51.7)	26 (40.6)	49 (54.4)	47 (59.5)	29 (37.7)	98 (57)	
**Total**	**482**	64 (100)	90 (100)	79 (100)	77 (100)	172 (100)	

*p < 0.05 = significant (Chi-square test).

Bold values to indicate statistical significance.

### 3.2 Prevalence and distribution of UTI

**Figure 1:**
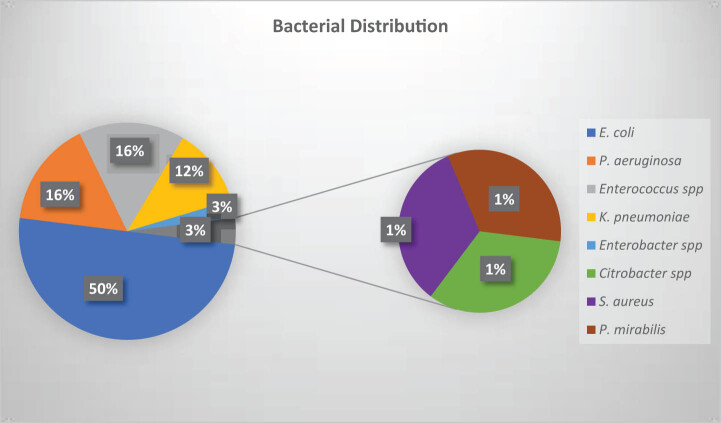
Distribution of bacteria causing UTIs among CKD patients.

The overall prevalence of UTI among CKD patients was 76/482 (15.8%). Patients with the age of >75 years (41.8%) and CKD stage 4 (33.8%) patients were found to be significantly associated with bacterial growth causing UTI (Table 2). Among the total 76 bacterial isolates, most were *E. coli* 50% (38/76), followed by *P. aeruginosa* 15.80% (12/76), *Enterococcus* spp. 15.80% (12/76), *Klebsiella pneumoniae* 11.84% (9/76), *Enterobacter* spp. 2.63% (2/76), *Citrobacter* 1.31% (1/76), *S. aureus* 1.31% (1/76), and *Proteus mirabilis* 1.31% (1/76), as shown in Figure 1.

**Table 2:** Study variables and prevalence of UTI

Variables	Total, *n* (%)	UTI, *n* (%)		*p*-Value
		Positive *n* (%)	Negative *n* (%)	
**Age group**				
<15 years	8 (1.7)	0	8 (100)	0.000*
15–30 years	49 (10.2)	11 (22.4)	38 (77.6)	
31–45 years	118 (24.5)	6 (5.1)	112 (94.9)	
46–60 years	127 (26.3)	13 (10.2)	114 (89.8)	
61–75 years	125 (25.9)	23 (18.4)	102 (81.6)	
>75 years	55 (11.4)	23 (41.8)	32 (58.2)	
**Gender**				
Male	233 (48.3)	39 (16.7)	194 (83.3)	0.572
Female	249 (51.7)	37 (14.9)	212 (85.1)	
**CKD stages**				
Stage 1	64 (13.3)	0	64 (100)	0.000*
Stage 2	90 (18.7)	0	90 (100)	
Stage 3	79 (16.4)	9 (11.4)	70 (88.6)	
Stage 4	77 (15.9)	26 (33.8)	51 (66.2)	
Stage 5	172 (35.7)	41 (23.8)	131 (76.2)	

*p < 0.05 = significant (Chi-square test).

Bold values to indicate statistical significance.

### 3.3 Antibiotic susceptibility pattern of the bacterial isolates

**Figure 2:**
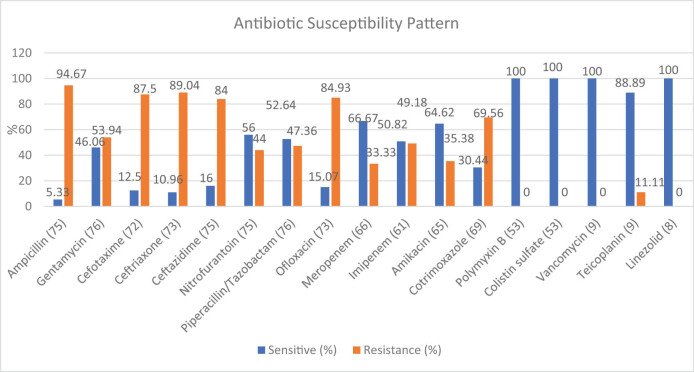
Antibiotic susceptibility pattern of bacterial isolates.

The majority of isolates were resistant to beta-lactam antibiotics, ampicillin (94.67%), ceftriaxone (89.04%), cefotaxime (87.5%), and ceftazidime (84.0%). Similarly, higher resistance was observed to ofloxacin (84.93%), cotrimoxazole (69.56%), and gentamycin (53.94%). However, all the tested gram-negative isolates were susceptible to polymyxin B and colistin. Similarly, all the tested GPB were sensitive to vancomycin and linezolid, as shown in Figure 2.

### 3.4 Resistance pattern of gram-negative isolates

Maximum numbers of gram-negative isolates were resistant to ampicillin, cefotaxime, ceftazidime, ceftriaxone, ofloxacin, and cotrimoxazole. At the same time, higher numbers of *E. coli* were sensitive to meropenem, amikacin, and nitrofurantoin, whereas *P. aeruginosa*, the most susceptible antibiotics were meropenem, amikacin, and piperacillin–tazobactam (PIT). Similarly, most *K. pneumoniae* were sensitive to meropenem, amikacin, PIT, and gentamycin (Table 3).

**Table 3:** Antibiotic resistance pattern of GNB (*N* = 63)

Antibiotics	Gram-negative bacterial isolates					
	*E. coli* (38)	*P. aeruginosa* (12)	*K. pneumoniae* (9)	*Enterobacter* spp. (2)	*Citrobacter* spp. (1)	*P. mirabilis* (1)
	%R	%R	%R	%R	%R	%R
Ampicillin	92.11 (35/38)	100 (11/11)	100 (9/9)	100 (2/2)	100 (1/1)	100 (1/1)
Gentamycin	47.37 (18/38)	66.67 (8/12)	44.44 (4/9)	50 (1/2)	0	0
Cefotaxime	86.48 (32/37)	81.82 (9/11)	77.78 (7/9)	100 (2/2)	100 (1/1)	100 (1/1)
Ceftriaxone	86.84 (33/38)	90.91 (10/11)	77.78 (7/9)	100 (2/2)	100 (1/1)	100 (1/1)
Ceftazidime	81.58 (31/38)	75 (9/12)	77.78 (7/9)	100 (2/2)	100 (1/1)	100 (1/1)
Nitrofurantoin	21.05 (8/38)	81.82 (9/11)	77.78 (7/9)	100 (2/2)	0	100 (1/1)
PIT	36.84 (14/38)	50 (6/12)	44.44 (4/9)	100 (1/1)	100 (1/1)	100 (1/1)
Ofloxacin	78.95 (30/38)	100 (11/11)	77.78 (7/9)	100 (1/1)	100 (1/1)	100 (1/1)
Meropenem	13.79 (4/29)	41.67 (5/12)	33.33 (3/9)	100 (1/1)	—	100 (1/1)
Imipenem	35.71 (10/28)	63.64 (7/11)	44.44 (4/9)	100 (1/1)	—	100 (1/1)
Amikacin	16.67 (5/30)	50 (6/12)	33.33 (3/9)	0	—	0
Cotrimoxazole	64.86 (24/37)	100 (8/8)	77.78 (7/9)	100 (1/1)	100 (1/1)	100 (1/1)
Polymyxin	0	0	0	0	—	0
Colistin	0	0	0	0	—	0

“–” = Not tested, %R = percentage resistance.

### 3.5 Resistance pattern of gram-positive isolates

Majority of *Enterococcus* species were resistant to cefotaxime (100%), ceftazidime (100%), ceftriaxone (100%), ofloxacin (100%), and ampicillin (91.67%). Vancomycin, linezolid, teicoplanin, nitrofurantoin, and cotrimoxazole were the most sensitive antibiotics against *Enterococcus* species. Similarly, *S. aureus* was resistant to all the tested antibiotics except meropenem, imipenem, nitrofurantoin, and PIT, as shown in Table 4.

**Table 4:** Antibiotic resistance pattern of GPB (*N* = 13)

Antibiotics	Gram-positive bacterial isolates	
	*Enterococcus* spp. (12)	*S. aureus* (1)
	%R	%R
Ampicillin	91.67 (11/12)	100 (1/1)
Gentamycin	75.00 (9/12)	100 (1/1)
Cefotaxime	100 (10/10)	100 (1/1)
Ceftriaxone	100 (10/10)	100 (1/1)
Ceftazidime	100 (11/11)	100 (1/1)
Nitrofurantoin	50.00 (6/12)	0
Piperacillin/tazobactam	75.00 (9/12)	0
Ofloxacin	100 (10/10)	100% (1/1)
Meropenem	66.67 (8//12)	0
Imipenem	77.78 (7/9)	0
Amikacin	81.82 (9/11)	—
Cotrimoxazole	50.00 (5/10)	100% (1/1)
Vancomycin	0	—
Teicoplanin	11.11 (1/9)	—
Linezolid	0	—

“–” = Not tested, %R = percentage resistance.

## 4 Discussion

CKD is considered a global public health burden, especially large in low- and middle-income countries [15]. Several risk factors, including hypertension, diabetes mellitus, glomerulonephritis, urolithiasis, and polycystic kidney disease, have been found to be linked to the development of kidney failure [16]. Patients with CKD and kidney failure may be at high-risk for infections, similar to patients with acquired immune deficiencies. In addition, chronic renal failure is a risk factor for UTIs due to metabolic disorders resulting in secondary immune alterations affecting many immune components [9]. Despite the lack of data on the prevalence of UTI in CKD patients, some researchers think it might be comparable to the general population [17]. This study established that the prevalence of bacteria causing UTI among CKD patients was reasonably high (15.8%) compared to the survey done by Almaiman et al. [18]. Different other studies have reported variable rates of UTI prevalent among CKD patients [19,20].

Our present study reported that the elderly population was associated with UTIs, and this result is consistent with research performed by Eshwarappa et al. and Manjunath et al. [5,21]. This result might be because elderly aged group were from advanced stages of CKD. Furthermore, the older age population showed urinary retention and excessive post-void residue, which is prone to the risk of UTIs. In addition, the most frequent causes of urinary stasis are prostate enlargement and autonomic neuropathy [22].

In this study, the prevalence of bacterial growth was high in males (16.7%) compared with the females (14.9%) though it was not statistically significant. In contrast to our findings, several previous studies documented the high prevalence of UTI in females compared with males [23,24]. This could be due to the proximity of the anus to the warm urethral tube in females. Furthermore, the urethral tube of the females is short, which shortens the distance the organism moves to the bladder [25]. These predisposing factors of UTI are accelerated by limited resources, poor hygiene, and low socioeconomic status [26]. However, a study by Deltourbe et al. reported that both sexes experience an equal rate of UTI among old people. Anatomical factors are inadequate to explain the UTI prevalence in an older male population because men’s urethral length does not alter with age. Instead, urodynamic alterations (such as prostatic enlargement in older men) or even cumulative biochemical or immunological abnormalities may be the reason for changes in the incidence of UTI in males [27]. Therefore, the male predominance of UTI in our study may be due to the higher percentage of elderly study participants.

Our study demonstrated *E. coli* as the most prevalent bacterial uropathogen, with 38/76 (50%). This finding is comparable with other studies elsewhere in Nepal, indicating 50–80% of isolation of *E. coli* [19,28,29]. *P. aeruginosa* and *Enterococcus* were the second most isolated uropathogen with 12/76 (16%) frequency each. The high frequency of *P. aeruginosa* and *Enterococcus* is unique to this study. Previous studies in Kathmandu (Nepal) 2021 and Dhaka (Bangladesh) 2022 reported low rates of *P. aeruginosa* (8.7 and 4.4%, respectively) and *Enterococcus* (9.9 and 6.4%, respectively) [30,31]. Although staphylococcal UTI was found to be more associated with increased use of bladder catheterization [23], our study reported lower rates of *S. aureus* (1%) isolated as uropathogen. Many other studies in different countries have reported a higher prevalence of *S. aureus* linked to UTI, from 5 to 15% [32,33,34]. The isolation of *K. pneumoniae* 9/76 (12%), *Enterobacter* 2/76 (3%), *Citrobacter* 1/76 (1%), and *P. mirabilis* 1/76 (1%) is in agreement with other studies by Odoki et al. in Uganda, Shakya et al. and Ganesh et al. from Nepal [23,30,35].

Antibiotics are crucial in treating bacterial infections as long as the etiological bacteria are susceptible to antibiotic activity. Thus, determining accurate antibiotic susceptibility is essential in treating bacterial infections [36]. Bacteria capable of acquiring resistance demand more attention as antibiotic resistance is a growing concern in low and middle-low-income countries [30]. In our study, maximum numbers of bacterial isolates were resistant to ampicillin (94.67%), cefotaxime (87.5%), ceftriaxone (89.04%), ceftazidime (84.0%), ofloxacin (84.93%), and cotrimoxazole (69.56%), which is similar to the study by Shankar et al. [2]. Among the gram-negative isolates, majority of *E. coli* were found to be resistant to ampicillin (92.11%), cephalosporins (cefotaxime [86.48%], ceftriaxone [86.84%], ceftazidime [81.58%]) and ofloxacin (78.95%). Polymyxin b, colistin, meropenem, amikacin, and nitrofurantoin were the most susceptible antibiotics to *E. coli*. *P. aeruginosa* and *K. pneumoniae* showed similar resistivity as they were also conferred highest resistance to ampicillin, cephalosporins, cotrimoxazole, and ofloxacin. This antibiotic susceptibility result of gram-negative isolates is concurrent with the findings of research performed by Shakya et al. and Ganesh et al. [30,35]. The possible reason for the higher resistance toward commonly used antibiotics might be inappropriate use and under-dose administration in the community. Also, horizontal resistance gene transfer from the multidrug resistant isolates in hospitals and communities might increase resistance in these isolates [37].

*Enterococcus* 15.8% (12/76) was the prevalent GPB in our study, whereas *S. aureus* was with minimal prevalence of 1% (1/76). Maximum numbers of *Enterococcus* were resistant to cefotaxime (100%), ceftriaxone (100%), ceftazidime (100%), ofloxacin (100%), ampicillin (91.67%), amikacin (81.82%), and gentamycin (75%). However, the most sensitive antibiotics were vancomycin, linezolid, teicoplanin, nitrofurantoin, and cotrimoxazole. In *S. aureus*, all the isolates were resistant to tested antibiotics except nitrofurantoin, PIT, meropenem, and imipenem. In our study, vancomycin, linezolid, nitrofurantoin, and meropenem were the most susceptible antibiotics against the gram-positive isolates. These findings align with that reported by Bhargava et al. and Mohapatra et al. from India (2022) [38,39]. This similarity might result from adopting similar antibiotic treatment recommendations for GPB in Nepal and India. Additionally, the species and strains of bacteria that are commonly found and cause diseases possibly are the reason for their concurrent resistance profile [6].

Despite being a single hospital-based study, our present study has reported some significant findings that might be useful for clinicians and urologists to choose appropriate antibiotics. However, a nationwide surveillance study is needed among CKD patients in Nepal to generalize these results. In addition, we only analyzed the phenotypic characteristics of the bacteria. Therefore, all the findings need to be confirmed by advanced genotypic methods.

## 5 Conclusion

There was a significant association between the elderly age group, CKD severity, and growth positivity for UTIs. In this study, GNB were more prevalent than GPB. *E. coli* was the predominant bacteria responsible for UTI among CKD patients. All the isolated bacteria showed higher resistance to commonly prescribed antibiotics in UTIs. This study recommends that, as CKD patients are immunocompromised, they can easily acquire the disease, and these resistant isolates can further worsen their health condition. To reduce the complicated infections and economic burden to CKD patients, routinely diagnosis of bacterial growth and their antibiogram would assist to prevent the burden of such infections. Therefore, our findings could help choose the correct antibiotics option to treat UTI among CKD patients.

## Abbreviations

AMR: antimicrobial resistance
CKD: chronic kidney disease
PIT: piperacillin–tazobactam
UTI: urinary tract infection
